# Identification of Target Genes of Antiarrhythmic Traditional Chinese Medicine Wenxin Keli

**DOI:** 10.1155/2020/3480276

**Published:** 2020-05-27

**Authors:** Yusi Yao, Yuhong Liu, Zhihuan Zeng, Yanqun Zhao, Tudi Li, Rong Chen, Rendan Zhang

**Affiliations:** Department of Cardiovascular Diseases, The First Affiliated Hospital of Guangdong Pharmaceutical University, Guangzhou, Guangdong 510080, China

## Abstract

Wenxin Keli (WXKL) is a traditional Chinese medicine drug approved for the treatment of cardiovascular diseases. This study aimed to identify WXKL-targeting genes involved in antiarrhythmic efficacy of WXKL. The Traditional Chinese Medicine Systems Pharmacology (TCMSP) technology platform was used to screen active compounds of WXKL and WXKL-targeting arrhythmia-related genes. A pig model of myocardial ischemia (MI) was established by balloon-expanding the endothelium of the left coronary artery. Pigs were divided into the model group and WXKL group (*n* = 6). MI, QT interval, heart rate, and arrhythmia were recorded, and the mRNA expression of target genes in myocardial tissues was detected by PCR. Eleven active ingredients of WXKL and eight WXKL-targeting arrhythmia-related genes were screened. Five pathways were enriched, and an “ingredient-gene-path” network was constructed. WXKL markedly decreased the incidence of arrhythmia in the MI pig model (*P* < 0.05). The QT interval was significantly shortened, and the heart rate was slowed down in the WXKL group compared with the model group (*P* < 0.05). In addition, the expression of sodium channel protein type 5 subunit alpha (*SCN5A*) and beta-2 adrenergic receptor (*ADRB2*) was downregulated, while muscarinic acetylcholine receptor M2 (*CHRM2*) was upregulated in the WXKL group (*P* < 0.05). In conclusion, WXKL may shorten the QT interval and slow down the heart rate by downregulating *SCN5A* and *ADRB2* and upregulating *CHRM2* during MI. These findings provide novel insight into molecular mechanisms of WXKL in reducing the incidence of ventricular arrhythmia.

## 1. Introduction

Acute occlusion of the epicardial coronary artery leads to myocardial ischemia (MI) with a rapid onset of unstable electrocardiograph (ECG) activity, which usually induces fatal ventricular arrhythmias [1]. Wenxin Keli (WXKL) is the first traditional Chinese medicine (TCM) approved as an antiarrhythmic drug by China Food and Drug Administration. A meta-analysis showed that WXKL was effective in the treatment of cardiovascular diseases (angina, heart failure, and arrhythmia), although more high-quality evidence was needed to support its use in clinical settings [2]. Compared with Western medicine treatment alone, combined use with WXKL could lower the heart rate, reduce the occurrence of arrhythmia (ventricular premature beats, ventricular tachycardia, and ventricular fibrillation), and improve heart function [3, 4]. Moreover, WXKL significantly reduced ventricular arrhythmia after MI [5]. Additionally, a recent study reported that WXKL could relieve recent-onset atrial fibrillation, without significant difference in the efficacy on male or female patients [6].

TCMs are oral preparations and need to reach target organs and tissues through the absorption, distribution, metabolism, and excretion (ADME) process. The ADME process plays a role in oral bioavailability (OB) and drug-likeness (DL), two pharmacokinetic characteristics of TCMs [7]. OB refers to the relative amount and rate at which the drug is absorbed into blood circulation after oral administration. DL refers to the similarity between the compound and the known listed drug. Compounds with OB ≥30% and DL ≥0.18 are considered potential active compounds for further analysis [8]. The TCM Systems Pharmacology (TCMSP) technology platform, which contains 499 herbs and their 29,000 chemical constituents, provides data on ADME properties of each compound, such as blood-brain barrier permeability, OB, and Caco-2 cell permeability, as well as targets for potentially active molecules (including 6,511 drug molecules in the DrugBank database and 3,987 proteins that interact with known compounds) and related disease information [9, 10]. The active components of WXKL and the targets related to arrhythmia can be retrieved from the platform. However, the effect of WXKL on cardiac electrical activity after MI and the mechanism of action of WXKL remain unclear.

The aim of this study was to investigate the effects of WXKL on arrhythmia and ECG activities after MI and identify WXKL-targeting genes involved in arrhythmia using TCMSP. We further confirmed WXKL-targeting genes in the animal model of MI.

## 2. Methods

### 2.1. Screening of Active Ingredients and Targets of WXKL

TCMSP was screened with “herb name” as the search item including the five ingredients of WXKL: *Nardostachys chinensis* Batal, *Codonopsis*, notoginseng,*Ambrum*, and rhizoma polygonati; OB was set to “Is greater than or equal to 30%”; DL was set to “Is greater than or equal to 0.18.”

### 2.2. KEGG Enrichment Analysis and Construction of the “Ingredient-Gene-Path” Network

Target genes were analyzed for KEGG pathway enrichment using DAVID (the Database for Annotation, Visualization, and Integrated Discovery; https://david.ncifcrf.gov/) v6.8. The target genes were directly mapped to the pathway, the number of genes was proportional to the significance of pathway enrichment, and the pathway of drug target enrichment was considered the pathway of drug regulation. The corresponding ingredients, genes, and pathways were constructed into a network diagram through Cytoscape 3.0 software.

### 2.3. Experimental Animals

All animal procedures were performed in accordance with the protocols approved by the Animal Care and Use Committee of Guangdong Pharmaceutical University (Guangzhou, China). Male miniature pigs (20–25 kg) were supplied by Guangdong Medical Laboratory Animal Center (Guangzhou, China) and randomized into two groups (*n* = 6): model group and WXKL group. In the WXKL group, WXKL purchased from Shandong Buchang Pharmaceuticals (8 g/kg, qd, mixed in the feed) was administered for 3 weeks prior to surgery. In the MI model, the pigs were anesthetized by injection with pentobarbital sodium (30 mg/kg) into the right common carotid artery, and the anterior descending branch of the left coronary artery (LAD) was expanded with interventional techniques. Then, 6*F* (1*F* = 0.33 mm) artery sheath tubes (Terumo Corporation, Japan) were introduced by guide wires through the iliac artery and placed in the left coronary artery under the C-arm X-ray machine (Artis zee III ceiling, Siemens, Germany). The balloon (Cordis; balloon : tube diameter = 1.3 : 1) entered the middle of the LAD via the guide wire and was inflated for 303.975 kPa for 30 s, repeated 3 times. The ECG monitor (Ruike Biotech, China) was used to continuously monitor the intraoperative and the postoperative electrocardiogram. The duration of QT, ST-T segment change, T-wave voltage, heart rate, and incidence of arrhythmia were recorded.

### 2.4. Quantitative PCR

The pigs were sacrificed, and myocardial tissue was removed and quickly frozen in liquid nitrogen and stored at −70°C. Total RNA was extracted from the tissue using the TRIzol reagent (Invitrogen). cDNA was synthesized from RNA using the RT kit (DBI, USA), and PCR was performed using the PCR kit (Genecopoeia, USA) and the following primers: SCN5A GGATTGTAGCTCCTCTCACTTC and GGAAGGCATCACTCTCTTCTAC; KCNH2 GAGATCGCATTCTACCGGAAAG and CTTCTCCATCACCACCTCAAAG; CHRM2 GCCTGCTATGCACTTTGTAATG and TCCTCTTGACTACCTTCCTTCT; ADRB2 CTCTTCCATCGTGTCCTTCTAC and CCTCAGACTTGTCGATCTTCTG; ADRB1 TCCGTCGTCTCCTTCTATGT and CGCAGCTGTCGATCTTCTT; ADRA1D GCAGACGGTCACCAACTATT and ACCTCCATAGTGGCAGAGAA; and GAPDH CAGGTTGTGTCCTGTGACTT and TTGACGAAGTGGTCGTTGAG. The expression levels of target genes were normalized to GAPDH and calculated using the 2−ΔΔCt method.

### 2.5. Statistical Analysis

Statistical analysis was conducted using the SPSS 21.0 software. The Mann–Whitney *U* test was used for all data analysis. Statistical significance was defined as *P* < 0.05.

## 3. Results

### 3.1. Arrhythmia-Related Target Genes of Active Ingredients of WXKL

Eleven active ingredients of WXKL were retrieved based on OB and DL. A total of eight WXKL-target genes related to arrhythmia were retrieved from TCMSP, including sodium channel protein type 5 subunit alpha (*SCN5A*), potassium voltage-gated channel subfamily H member 2 (*KCNH2*), beta-1 adrenergic receptor (*ADRB1*), beta-2 adrenergic receptor (*ADRB2*), alpha-1D adrenergic receptor (*ADRA1D*), muscarinic acetylcholine receptor M2 (*CHRM2*), alpha-2A adrenergic receptor (*ADRA2A*), and gap junction alpha-1 protein (*GJA1*) (Table 1).

### 3.2. I-G-P Network Construction and Analysis

**Figure 1:**
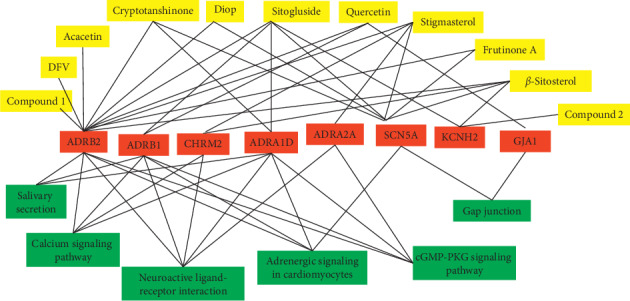
Ingredient-gene-path (I-G-P) network for the ingredients of WXKL, drug-target genes, and KEGG pathways. Yellow nodes represent eleven active compounds, red nodes represent eight targets, and green nodes represent enriched signal pathways.

Based on I-G-P network construction, we established the interactions among eleven active compounds of WXKL, eight targets, and six enriched KEGG pathways. *ADRB2* and *SCN5A* were the targets of the most ingredients, and calcium signaling pathway, neuroactive ligand-receptor interaction, adrenergic signaling in cardiomyocytes, and cGMP-PKG signaling pathway were the most important signaling pathways that may mediate antiarrhythmic effects of WXKL (Figure 1).

### 3.3. Electrophysiological and Antiarrhythmic Effects of WXKL

ST-segment abnormalities (different levels of T-wave low-level, inverted, ST-segment elevation) occurred after the balloon dilated the coronary artery, and all pigs developed ventricular tachycardia 3–6 minutes after the balloon began to expand, and all pigs had ventricular fibrillation about 5–7 minutes after expansion. Postoperative heart rates of both groups were significantly increased (all *P* < 0.05), but heart rates were significantly slower in the WXKL group compared to the model group (*P* < 0.05). There was no statistical difference in the QT interval before and after surgery, but the QT interval in the model group was significantly longer than that in the WXKL group (*P* < 0.05) (Table 2). In addition, the incidence of heart dysfunction was significantly lower in the WXKL group compared to the model group (*P* < 0.05) (Table 3). These data indicated the antiarrhythmic effects of WXKL.

### 3.4. WXKL Regulated the Expression of Targets in Myocardial Tissue

**Figure 2:**
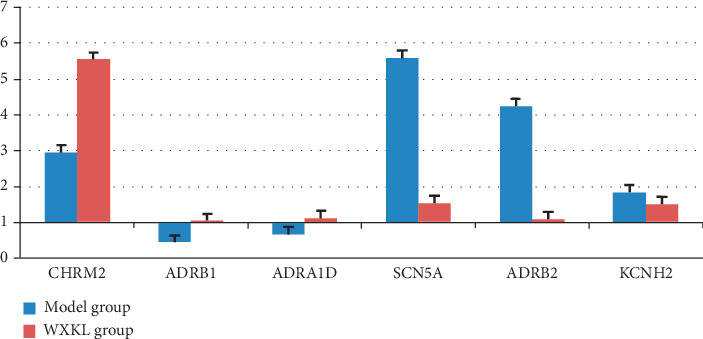
Effects of WXKL on *CHRM2*, *ADRB1*, *ADRA1D*, *SCN5A*, *ADRB2*, and *KCNH2* mRNA expression levels during MI.

Finally, we selected 6 targets and examined the effects of WXKL on their expression in myocardial tissues of the animal models. PCR analysis showed that *SCN5A* and *ADRB2* mRNA levels were significantly lower and the *CHRM2* mRNA level was significantly higher in the WXKL group than in the model group, *KCNH2* and *ADRA1D* expressions showed no significant difference between two groups, while *ADRB1* was not expressed in two groups (Figure 2).

## 4. Discussion

*ADRB1*, *ADRB2*, *ADRA1D*, and *ADRA2A* are G-protein-coupled transmembrane receptors that mediate sympathetic nervous system activity by binding neurotransmitters such as catecholamines, epinephrine, and norepinephrine [11, 12]. CHRM2 binds to acetylcholine to mediate the activity of the parasympathetic nervous system. Cardiac autonomic nerves can induce or promote arrhythmias directly or indirectly by altering electrophysiological features [13, 14]. A large retrospective study demonstrated that beta blockers and ACE inhibitors are associated with improved secondary survival in patients surviving ventricular arrhythmias on admission [15]. KCNH2 mediates the rapid activation of delayed rectifier potassium current (IKr), which is important for normal ventricular repolarization [16]. *SCN5A* encodes voltage-gated sodium channel 1.5 (Nav1.5), which mediates inward sodium current (INa) and induces rapid depolarization. *SCN5A* mutations can impair the function of Nav1.5 and induce various arrhythmias [17]. The cardiomyocyte gap junction is the structural basis for the diffusion of action potentials in myocardial tissue and plays an important role in cell electrical coupling and action transmission [18]. The major gap junction of ventricular myocytes is GJA1 (*Cx43*). Cardiac pathological conditions affect the expression, translocation, and distribution of the gap junction, interfere with communication between cardiomyocytes, increase the cardiac potential decoupling rate, and induce arrhythmia [19]. Wen et al. reported that WXKL prevented ventricular arrhythmias induced by myocardial ischemia-reperfusion by upregulating the expression of *Cx43* [5].

The QT interval represents the depolarization and repolarization time of the ventricle, and the prolongation of the QT interval is important for malignant arrhythmias and sudden cardiac death [20]. WXKL is an effective alternative to prevent potentially fatal arrhythmias after myocardial infarction in an animal model [21]. In this study, ECG showed that WXKL could significantly shorten the QT interval, slow down the heart rate after MI, and reduce the incidence of arrhythmia, consistent with the results summarized in a recent review [22].

An early study reported that WXKL exerted antiarrhythmic effects by selectively inhibiting sodium current [23]. *SCN5A* gene mutation in patients with long QT syndrome delayed the sodium channel closure and increased myocardial cell potential, which prolonged the 2-phase plateau of action potential, leading to prolongation of the QT interval [24]. These results indicate that WXKL may lead to downregulation of the expression of *SCN5A* and inhibition of sodium inward currents.

Adrenaline may increase myocardial repolarization dispersion by acting on the *β*2 receptor, triggering arrhythmia [25]. The use of *β*2 receptor antagonists significantly reduced the incidence of ventricular fibrillation [26]. Consistent with these results, in this study, we found that WXKL selectively decreased the expression of the *β*2 receptor, in which WXKL may reduce the incidence of arrhythmia after MI by downregulating the *β*2 receptor. Activation or overexpression of the *β*2 receptor stimulates the L-calcium channel, resulting in a significant increase in L-type Ca^2+^ current. Within a few minutes from coronary occlusion, the initial decrease in the duration and amplitude of the cardiac electrical potential at rest occurs. This is due to ischemia-induced decrease of inward sodium currents with upregulation of calcium inward currents, causing initially prolonged and finally shorter QT interval. Moreover, some “border zones” between ischemic and nonischemic areas create areas with different refractory periods, which, along with acidosis (caused by ischemia), damage of the “gap junctions,” and impaired conduction, lead to possible reentry circuits which, actually, may account for the most clinical relevant arrhythmias in ischemic heart disease. Indeed, Wang et al. found that WXKL may attenuate myocardial ischemia-induced arrhythmias by inhibiting L-calcium current and transient outward potassium current [27]. In addition, the QT interval can be affected by inhibiting L-calcium current [28]. We supposed that, after myocardial ischemia, WXKL may inhibit L-type calcium channels by downregulating the expression of the *β*2 receptor, which in turn is involved in the regulation of the QT interval.

Timely correction of tachycardia during myocardial ischemia is important for preventing arrhythmias, and blocking the acetylcholine M receptor leads to faster heart rates [29]. CHRM2 is involved in the regulation of the heart rate [30]. Our results showed that WXKL significantly increased the mRNA expression of CHRM2, suggesting that WXKL could slow down the heart rate by upregulating CHRM2.

In conclusion, the network diagram showed that SCN5A and ADRB2 were the main targets for most active ingredients of WXKL, which are consistent with the important role of SCN5A and ADRB2 in the regulation of cardiac function. In summary, we screened potential targets of active ingredients of WXKL involved in the regulation of arrhythmia through TCMSP and confirmed that WXKL could shorten the QT interval and slow down the heart rate by downregulating *SCN5A* and *ADRB2* and upregulating *CHRM2* during MI. These findings provide novel insight into potential molecular mechanisms of WXKL in affecting cardiac electrical activation. Further investigation is actually required for a better definition of the role of WXKL in ischemia-induced changes in the different ion channels, as well.

## Figures and Tables

**Table 1:** Arrhythmia-related targets of active ingredients of WXKL.

Compound	OB	DL	Target gene
Acacetin	34.97	0.24	*ADRB2*
Cryptotanshinone	52.34	0.4	*ADRB2*/*SCN5A*/*ADRA1D*
Stigmasterol	43.83	0.76	*ADRB2*/*ADRB1*/*SCN5A*/*CHRM2*/*ADRA2A*
Quercetin	46.43	0.28	*ADRB2*/*GJA1*
Sitogluside	20.63	0.62	*ADRB2*/*ADRB1*/*KCNH2*/*SCN5A*/*ADRA1D*
*β*-Sitosterol	36.91	0.75	*ADRB2*/*KCNH2*/*SCN5A*/*CHRM2*
Diop	43.59	0.39	*ADRB2*/*SCN5A*
Frutinone A	65.9	0.34	*ADRB2*/*SCN5A*
Compound 1	32.16	0.41	*ADRB2*
DFV	32.76	0.18	*ADRB2*
Compound 2	71.12	0.18	*KCNH2*

Compound 1: 3-beta-hydroxymethylenetanshiquinone. Compound 2: (2R)-7-hydroxy-2-(4-hydroxyphenyl)chroman-4-one.

**Table 2:** QT interval and heart rate effects of WXKL.

Group	*n*	Preoperative		Postoperative	
		QT (ms)	HR (bpm)	QT (ms)	HR (bpm)
Model group	6	433.33	88.5	436.50	120.50
		(419.10–439.90)	(78.00–99.00)	(408.30–453.36)	(112.00–134.00)

WXKL group	6	422.20	89.00	406.15^*∗*^	103.00^*∗*^
		(337.90–438.60)	(73.00–98.00)	(318.90–425.30)	(85.00–120.00)

All data are expressed as the median, maximum, and minimum; ^*∗*^*P* < 0.05 compared with the model group and preoperation.

**Table 3:** Comparison of the incidence of ventricular arrhythmias.

Group	*n*	VT	VF	Incidence (%)
Model group	6	2	2	4 (67)
WXKL group	6	2^*∗*^	0^*∗*^	2 (33)

VT: ventricular tachycardia; VF: ventricular fibrillation; ^*∗*^*P* < 0.05 compared with the model group.

## Data Availability

All data used to support the findings of this study are available from the corresponding author upon request.

## References

[1] Houshmand F., Faghihi M., Imani A., Kheiri S. (2017). Effect of different doses of oxytocin on cardiac electrophysiology and arrhythmias induced by ischemia. *Journal of Advanced Pharmaceutical Technology & Research*.

[2] Wang X., Wang Y., Feng X. Y. (2016). Systematic review and meta-analysis of randomized controlled trials on Wenxin keli. *Drug Design, Development and Therapy*.

[3] Zheng R., Tian G. H., Zhang Q., Wu L., Xing Y., Shang H. (2018). Clinical safety and efficacy of Wenxin keli-amiodarone combination on heart failure complicated by ventricular arrhythmia: a systematic review and meta-analysis. *Frontiers Physiol*.

[4] Montes F. O., Vaquez-Hernadez A., Fenton-Navarro B. (2019). Active compounds of medicinal plants, mechanism for antioxidant and beneficial effects. *Phyton-International Journal of Experimental Botany*.

[5] Wen Z., Jiao Z. Y., Fei L. X., Jian S. (2014). GW25-e4276 the mechanism of Wenxin Granule prevention on ventricular arrhythmia during acute myocardial ischemia and reperfusion. *Journal of the American College of Cardiology*.

[6] Zhang N., Tse G., Dahal S. (2018). Efficacy of Wenxin keli plus amiodarone versus amiodarone monotherapy in treating recent-onset atrial fibrillation. *Cardiology Research and Practice*.

[7] Hou T., Xu X. (2002). ADME evaluation in drug discovery. *Journal of Molecular Modeling*.

[8] Veber D. F., Johnson S. R., Cheng H.-Y., Smith B. R., Ward K. W., Kopple K. D. (2002). Molecular properties that influence the oral bioavailability of drug candidates. *Journal of Medicinal Chemistry*.

[9] Ru J., Li P., Wang J. (2014). TCMSP: a database of systems pharmacology for drug discovery from herbal medicines. *Journal of Cheminformatics*.

[10] Villa-Hernández J. M., García-Ocón B., Sierra-Palacios E. C., Pelayo-Zaldivar C. (2018). Molecular biology techniques as new alternatives for medicinal plant identification. *Phyton-International Journal of Experimental Botany*.

[11] Tatiana L. F., Vanda J., Cristiane C. C. (2015). Double disruption of *α*2A- and *α*2C-adrenoceptors results in sympathetic hyperactivity and high-bone-mass phenotype. *Journal of Bone and Mineral Research*.

[12] Perez D. M., Doze V. A. (2011). Cardiac and neuroprotection regulated by *α*1-adrenergic receptor subtypes. *Journal of Receptors and Signal Transduction*.

[13] Scherlag B. J., Nakagawa H., Jackman W. M. (2005). Electrical stimulation to identify neural elements on the heart: their role in atrial fibrillation. *Journal of Interventional Cardiac Electrophysiology*.

[14] Charles C. J., Jardine D. L., Richards A. M. (2007). Cardiac sympathetic nerve activity and ventricular fibrillation during acute myocardial infarction in a conscious sheep model. *American Journal of Physiology. Heart and Circulatory Physiology*.

[15] Schupp T., Behnes M., Weiß C. (2018). Beta-blockers and ACE inhibitors are associated with improved survival secondary to ventricular tachyarrhythmia. *Cardiovascular Drugs Therapy*.

[16] Schroder E. A., Burgess D. E., Zhang X. (2015). The cardiomyocyte molecular clock regulates the circadian expression of Kcnh2 and contributes to ventricular repolarization. *Heart Rhythm*.

[17] Amin A. S., Reckman Y. J., Arbelo E. (2018). *SCN5A* mutation type and topology are associated with the risk of ventricular arrhythmia by sodium channel blockers. *International Journal of Cardiology*.

[18] Gao J., Zhao Y., Wang Y. (2015). Anti-arrhythmic effect of acupuncture pretreatment in the rats subjected to simulative global ischemia and reperfusion-involvement of intracellular Ca2+ and connexin 43. *BMC Complementary and Alternative Medicine*.

[19] Alessandra R., Martina C., Jolanda V. H., Frans V. R. (2014). Intercalated discs and arrhythmogenic cardiomyopathy. *Circulation Cardiovascular Genetics*.

[20] Polyák A., Kui P., Morvay N. (2018). Long-term endurance training-induced cardiac adaptation in new rabbit and dog animal models of the human athlete’s heart. *Reviews in Cardiovascular Medicine*.

[21] Wu A., Lou L., Zhai J. (2017). Effect of Wenxin granules on gap junction and MiR-1 in rats with myocardial infarction. *BioMed Research International*.

[22] Tian G., Sun Y., Liu S. (2018). Therapeutic effects of Wenxin keli in cardiovascular diseases: an experimental and mechanism overview. *Frontiers in Pharmacology*.

[23] Hou J.-W., Li W., Guo K. (2016). Antiarrhythmic effects and potential mechanism of WenXin KeLi in cardiac purkinje cells. *Heart Rhythm*.

[24] Han D., Tan H., Sun C., Li G. (2018). Dysfunctional Nav1.5 channels due to *SCN5A* mutations. *Experimental Biology and Medicine*.

[25] Adameova A. D., Bhullar S. K., Elimban V., Dhalla N. S. (2018). Activation of *β*1-adrenoceptors may not be involved in arrhythmogenesis in ischemic heart disease. *Reviews in Cardiovascular Medicine*.

[26] Liang D., Wu Y., Zhou L. (2019). LRP5 controls cardiac QT interval by modulating the metabolic homeostasis of L-type calcium channel. *International Journal of Cardiology*.

[27] Wang X., Wang X., Gu Y., Wang T., Huang C. (2013). Wenxin Keli attenuates ischemia-induced ventricular arrhythmias in rats: involvement of L-type calcium and transient outward potassium currents. *Molecular Medicine Reports*.

[28] Guo D., Zhou J., Zhao X. (2008). L-type calcium current recovery versus ventricular repolarization: preserved membrane-stabilizing mechanism for different QT intervals across species. *Heart Rhythm*.

[29] Greenberg S., Plummer C., Maisenbacher H., Friary J., Berg A. (2015). The effect of topical ophthalmic 1% atropine on heart rate and rhythm in normal dogs. *Veterinary Ophthalmology*.

[30] Bergfeldt L., Lundahl G., Bergqvist G., Vahedi F., Gransberg L. (2017). Ventricular repolarization duration and dispersion adaptation after atropine induced rapid heart rate increase in healthy adults. *Journal of Electrocardiology*.

